# Identification of key genes involved in collagen hydrogel-induced chondrogenic differentiation of mesenchymal stem cells through transcriptome analysis: the role of m6A modification

**DOI:** 10.1007/s10856-024-06801-2

**Published:** 2024-07-29

**Authors:** Chaotao Chen, Kai Xiong, Kanglu Li, Bo Zhou, Jianwen Cheng, Bo Zhu, Li Zheng, Jinmin Zhao

**Affiliations:** 1 Guangxi Engineering Center in Biomedical Materials for Tissue and Organ Regeneration, The First Affiliated Hospital of Guangxi Medical University, Guangxi Medical University, Nanning, 530021 China; 2 Collaborative Innovation Centre of Regenerative Medicine and Medical BioResource Development and Application Co-constructed by the Province and Ministry, Guangxi Medical University, Nanning, 530021 China; 3 Department of Orthopaedics Trauma and HandSurgery, The First Affiliated Hospital of Guangxi Medical University, Guangxi Medical University, Nanning, 530021 China; 4 Department of Orthopaedics Trauma and HandSurgery, The Third Affiliated Hospital of Guangxi Medical University, Guangxi Medical University, Nanning, 530031 China; 5 International Joint Laboratory of Ministry of Education for Regeneration of Bone and Soft Tissues, The First Affiliated Hospital of Guangxi Medical University, Guangxi Medical University, Nanning, 530021 China; 6 Guangxi Key Laboratory of Regenerative Medicine, The First Affiliated Hospital of Guangxi Medical University, Guangxi Medical University, Nanning, 530021 China

## Abstract

Collagen hydrogel has been shown promise as an inducer for chondrogenic differentiation of bone marrow mesenchymal stem cells (BMSCs), contributing to the repair of cartilage defects. However, the precise molecular mechanism underlying this phenomenon remains poorly elucidated. Here, we induced chondrogenic differentiation of BMSCs using collagen hydrogel and identified 4451 differentially expressed genes (DEGs) through transcriptomic sequencing. Our analysis revealed that DEGs were enriched in the focal adhesion pathway, with a notable decrease in expression levels in the collagen hydrogel group compared to the control group. Protein-protein interaction network analysis suggested that actinin alpha 1 (ACTN1) and actinin alpha 4 (ACTN4), two proteins also involved in cytoskeletal recombination, may be crucial in collagen hydrogel-induced chondrogenic differentiation of BMSCs. Additionally, we found that N6-methyladenosine RNA methylation (m6A) modification was involved in collagen hydrogel-mediated chondrogenic differentiation, with fat mass and obesity-associated protein (FTO) implicated in regulating the expression of ACTN1 and ACTN4. These findings suggest that collagen hydrogel might regulate focal adhesion and actin cytoskeletal signaling pathways through down-regulation of ACTN1 and ACTN4 mRNA via FTO-mediated m6A modification, ultimately driving chondrogenic differentiation of BMSCs. In conclusion, our study provides valuable insights into the molecular mechanisms of collagen hydrogel-induced chondrogenic differentiation of BMSCs, which may aid in developing more effective strategies for cartilage regeneration.

**Graphical Abstract:**

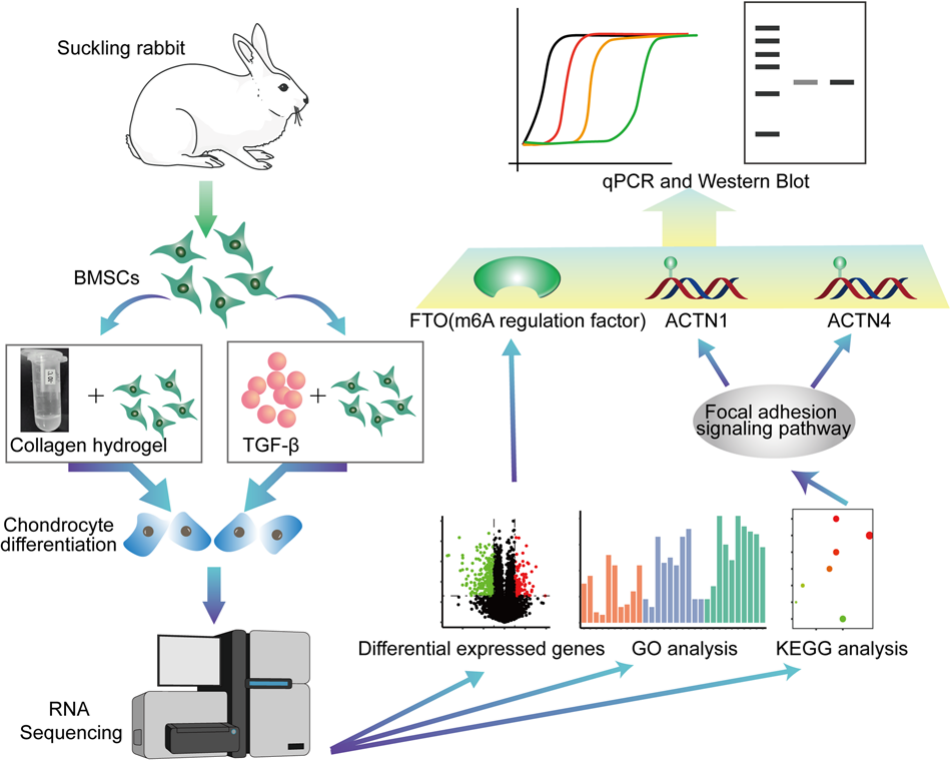

## Introduction

Articular cartilage damage is a common problem in orthopedics, usually caused by trauma or chronic repetitive overload. It is often accompanied by severe discomforts such as pain, swelling, and functional limitation. Articular cartilage is a type of connective tissue that lacks blood vessels and nerves. This unique characteristic limits its intrinsic ability to self-repair, making it challenging to regenerate damaged tissue. Thus, the restoration of articular cartilage defects poses a significant clinical challenge in the orthopedic field. The current clinical approaches for repairing and regenerating damaged articular cartilage, such as bone marrow stimulating techniques (microfracture), osteochondral transfer, and autologous chondrocyte implantation, have shown some success in treating these defects. Unfortunately, these methods often result in unpredictable outcomes and require multiple surgeries, which are insufficient for achieving optimal long-term cartilage regeneration results [1]. Hence, there is a pressing need for the development of innovative treatment approaches to address articular cartilage defects.

Bone marrow-derived mesenchymal stem cells (BMSCs) are a type of multipotent stromal cells that possess the ability to differentiate into diverse cell lineages, including chondrocytes, which are the cells responsible for producing and maintaining articular cartilage [2]. The chondrogenic differentiation potential of BMSCs has made them an attractive candidate for the management of articular cartilage defects. Recent research has shown that BMSCs have a great potential for differentiating into chondrocytes and have been widely investigated for cell-based therapy in cartilage repair [3]. Thus, enhancing the proliferation and chondrogenic differentiation of BMSCs is a crucial step in treating cartilage defects with BMSCs. However, inducing BMSCs to differentiate into chondrocytes is challenging and requires the use of appropriate growth factors or other inducers. Although traditional growth factors can effectively induce BMSCs differentiation into chondrocytes, they have several limitations that restrict their clinical application, such as rapid degradation, high cost, functional heterogeneity, and difficulty in achieving consistent results [4–7]. Therefore, it is necessary to find an economical and effective chondrogenic inducer.

Collagen hydrogel has been recognized as a promising material due to its cost-effectiveness, excellent biocompatibility, and ability to easily fill defects of almost any shape or size. In addition, collagen hydrogel mimics the natural ECM of cartilage and provides an environment that can promote cell attachment, proliferation, and differentiation [8, 9]. We previously demonstrated that collagen hydrogel effectively induced chondrogenic differentiation of BMSCs without adding any extraneous factors [10]. Furthermore, when compared to mesh or velvet scaffolds, which often only allow for cell implantation on the scaffold surface, chondrocytes embedded within the hydrogel are evenly dispersed, thus facilitating the uniform synthesis of extracellular matrix components immediately after transplantation [11]. As research on the topic continues to grow, collagen hydrogel is emerging as a promising material for treating articular cartilage defects by driving the chondrocyte differentiation of BMSCs. Nevertheless, the precise molecular mechanisms underlying collagen hydrogel-mediated regulation of chondrogenic differentiation remain largely elusive. The genes that are crucial for the process of chondrogenesis and how the material instigates the chondrogenic differentiation of BMSCs have not yet been fully determined.

M6A modification is an epigenetic process that exerts regulatory effects on gene expression and plays a crucial role in the progression of chondrogenic differentiation. Emerging evidence has highlighted the importance of m6A modification in regulating diverse biological processes, such as stem cell differentiation and development. For instance, Hu et al. [12] reported that METTL3, an m6A methyltransferase, was significantly involved in the chondrogenic differentiation of synovium-derived mesenchymal stem cells. Similarly, another study by Yang [13] et al. reported that YTHDF1, a main m6A reader protein, enhances the chondrogenic differentiation of human bone marrow mesenchymal stem cells. Therefore, investigating the molecular basis of collagen hydrogel-induced chondrogenesis and identifying key genes involved in m6A modification during this process may provide valuable information to guide the development of more effective strategies for inducing chondrogenic differentiation in BMSCs and ultimately improve the clinical outcomes of cartilage repair.

In this study, we aimed to identify critical genes implicated in m6A modification during collagen hydrogel-induced chondrogenic differentiation of BMSCs through transcriptomic analysis. Using RNA sequencing technology, we identified the differentially expressed genes of BMSCs during chondrogenic differentiation induced by collagen hydrogel under a three-dimensional system. Our focus was primarily on identifying the genes involved in m6A modification during the process of collagen hydrogel-induced chondrogenic differentiation. These findings shed light on the crucial genes and underlying intracellular mechanisms involved in chondrogenic differentiation, thereby advancing the potential application of collagen hydrogel in cartilage regeneration.

## Materials and methods

### Extracting and culturing of BMSCs

The rabbits employed in this study were sourced from the Medical Laboratory Animal Center, Guangxi Medical University. The animal operations were executed as directed by the guidelines outlined by the National Institutes of Health’s “Guide for the Care and Use of Laboratory Animals” (Eighth Edition) and approved by the Animal Ethic Committee of Guangxi Medical University. The BMSCs were extracted from the bone marrow of suckling rabbits at three to four days of age. Briefly, the limbs of suckling rabbits were placed on the ultra-clean bench to remove the attached soft tissue of the femur, cut off the epiphysis, and repeatedly irrigated the bone marrow cavity to obtain mononuclear cells. The cells were subsequently seeded into a six-well plate and maintained in Dulbecco’s modified Eagle’s medium (DMEM)/Basic (Hyclone) that had been supplemented with 10% fetal bovine serum (FBS, Gemini) as well as 100 μg/mL streptomycin and 100 units/mL penicillin (Solarbio, China). They were then incubated at 37 °C within an incubator from Thermo Fisher Scientific, UK, under optimal conditions of a humidified atmosphere that contained 5% CO_2_. After a period of seven days, the BMSCs were collected and sub-cultured into a 10 cm plastic dish at a suitable concentration (4 × 10^3^ cells/cm^2^). To ensure optimal cell viability and growth, the medium was refreshed every three days. When cells reached 100% confluency, the cultured cells were detached using the trypsin-EDTA solution containing 0.25% trypsin (Solarbio, China) and 0.02% EDTA and sub-cultured at a split ratio of 1:3. The BMSCs from the third passage were utilized for further research. The cells were allocated into three groups and exposed to various reagents. (1) The experimental group: the BMSCs were co-cultured with 100 μL of collagen hydrogel in a complete culture medium (CM). (2) The blank control group: the BMSCs were co-cultured with PBS in CM. (3) The TGF‐β group: the BMSCs were co-cultured with TGF‐β in CM. The cells in each group were harvested after 7 days and 14 days of incubation for further investigation.

### RNA sequencing analysis

To ensure the quality and integrity of total RNA prior to constructing the sequencing library, we employed agarose gel electrophoresis to assess its integrity and quantified it using NanoDrop ND-1000 (Thermo Scientific, Wilmington, DE, USA). 1–2 μg of total RNA was selected from each sample for the construction of the sequencing library. Enrichment of total RNA was achieved using either the NEB Next Poly(A) mRNA Magnetic Isolation Module or RiboZero Magnetic Gold Kit based on the manufacturer’s instructions. After treatment, we constructed the library using the KAPA Stranded RNA-Seq Library Prep Kit (Illumina), which utilizes dUTP-based chain-specific library construction techniques. The constructed libraries were inspected by using Agilent 2100 Bioanalyzer (Agilent Technology, United States) and quantified by qPCR. Subsequently, the sequencing libraries of different samples were subjected to denaturation by 0.1 M NaOH, leading to the generation of single-stranded DNA fragments. And then, the fragments were diluted into a concentration of 8 pM and underwent in situ amplification with the use of the TruSeq SR Cluster Kit v3-cBot-HS (#GD-401-3001, Illumina), after which they were sequenced on an Illumina HiSeq 4000 platform. The results were compared to the known transcriptome through the software StringTie, and the calculation of transcription abundance was performed using Ballgown, with the measurement unit for expression quantity being Fragments Per Kilobase of gene/transcript model per Million mapped fragments (FPKM).

### Identification of differentially expressed genes

Differentially expressed genes (DEGs) related to chondrogenic differentiation of BMSCs in the collagen hydrogel group versus the Blank control group were determined using the edgeR package from Bioconductor. The genes with a *p* value threshold below 0.05, as well as an absolute log2(fold-change) greater than 0.5, were known as DEGs.

### Enrichment analysis of differentially expressed genes on Kyoto Encyclopedia of Genes and Genomes and Gene Ontology

The R package clusterProfiler was utilized to accomplish enrichment analyses on Kyoto Encyclopedia of Genes and Genomes (KEGG) pathways and Gene Ontology (GO) biological processes. Enrichment was defined as a *p* value below 0.1 and a q-value below 0.2. Additionally, the “ggplot2” package was employed to enhance visualization of the connection between candidate genes and classical biological function and signaling pathway.

### Construction of PPI network

The genes of critical signaling pathways were screened to create protein-protein interaction (PPI) networks. Each node of the network was identified and visualized with the use of the CytoHubba plugin (version 3.9.1) in Cytoscape software. The top two proteins/genes with the highest number of connections were selected and designated as “hub” genes.

### Correlation analysis

In order to evaluate the association between m6A-related regulatory factors and essential genes, R programming was utilized to perform both Spearman’s and Pearson’s correlation tests as well as Fisher’s exact test. The aim of these tests was to determine the degree of correlation between the two groups of variables.

### Real-time quantitative polymerase chain reaction analysis

The HiPure total RNA Mini kit from Magen in Guangzhou, China, was used to isolate total RNA. And cDNA was synthesized using random primers and the Prime ScriptTM RT reagent kit from Takara in Tokyo, Japan. Targeted genes were selected and measured by the real-time quantitative polymerase chain reaction (RT-qPCR). Table 1 lists all gene-specific primer sequences used in the experiment. GAPDH served as the reference gene to standardize the quantity of cDNA in PCR reactions. The RT-qPCR was conducted in a LightCycler @ 96 PCR instrument from Roche in Basel, Switzerland, in accordance with the manufacturer’s instructions. SYBR® Primix Ex Taq II from Takara in Beijing, China, was utilized along with 1 μL of cDNA, 0.5 mol/L of each primer in a total volume of 20 μL. The RT-qPCR reaction was amplified by cycling 40 times through denaturation at 95 °C for 15 sec and annealing/extension at 60 °C for 0.5 min per cycle. The relative expression of RNA was calculated by applying the 2^-△△^Ct method, which involves quantifying the fold change in gene expression normalized to an internal control.

**Table 1:** The RT-qPCR primers utilized in this study

Gene	Sense (5’-3’)	Antisense (5’-3’)
GAPDH	TCCAGTATGACTCTACCCACG	CACGACATACTCAGCACCAG
ACTN1	CTTCGACAACAAGCACACCA	TCCCGATCGAAGTGGTTGAA
ACTN4	CTTCGACAACAAGCACACCA	ACCATGGTCCTTGTCGAAGT
FTO	GGCACTGCTGTCACTTTGAA	CTTCCACAGTCCCTCCAGTT

### Western Blot

The protein was extracted from cells by utilizing protein lysis buffer (RIPA). The protein samples obtained from various groups were separated using 10% sodium lauryl sulfate-polyacrylamide gel electrophoresis (SDS-PAGE) at 120 V for a duration of 45 min and subsequently transferred to polyvinylidene fluoride (PVDF) membranes (Biosharp, Beijing, China). These samples were then transferred onto polyvinylidene fluoride (PVDF) membranes (Biosharp, Beijing, China). To prevent non-specific binding, the membrane was blocked with skim milk before being incubated overnight at 4 °C with anti-ACTN1(ab68194, Abcam, UK), anti-ACTN4 (ab108198, Abcam, UK), anti-GAPDH (ab181602, Abcam, UK) and anti-FTO (ab126605, Abcam, UK) antibodies. Subsequently, the membrane was washed three times with PBS + 0.1% Tween 20 (PBST) to remove any unbound or non-specifically bound antibodies. The appropriate second HRP-conjugated antibody (D111018, Sangon Biotech, China) was used to incubate the membrane. Following this step, the membrane was washed three times using PBST to remove any unbound antibodies. Chemiluminescence (ECL) solutions and Amersham Imager 600 (General Electric, United States) were used to detect immune response bands, and ImageJ software was utilized to measure the intensity of each band. GAPDH was employed as an internal protein for normalization purposes. This test was conducted in triplicate.

### Statistical analysis

The data was analyzed using GraphPad Software (GraphPad Prism 8.0), with mean ± standard deviation (SD) of triplicate repeated experiments. For group comparisons, an unpaired Student’s *t* test was used, and *p* values less than 0.05 were regarded as statistically significant for all measurements.

## Results

### Global gene expression profile in the chondrogenic differentiation of BMSCs induced by collagen hydrogel and determination of DEGs

**Fig. 1:**
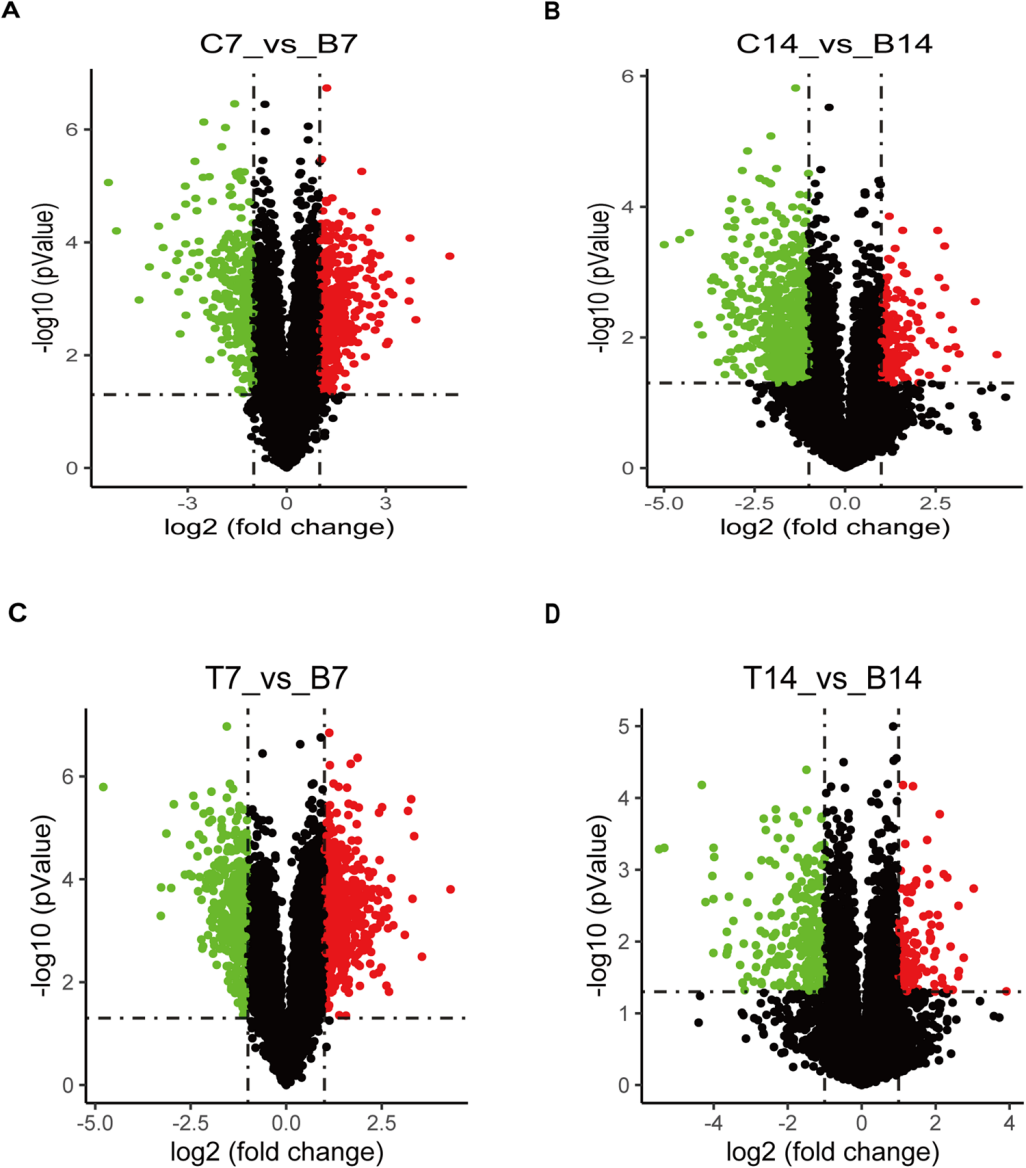
Volcano plot of screening differential expressed genes (DEGs). **A** Analysis of DEGs in C7 vs. B7 group; (**B**) Analysis of DEGs in C14 vs. B14 group; (**C**) Analysis of DEGs in T7 vs. B7 group; (**D**) Analysis of DEGs in T14 vs. B14 group. C: collagen hydrogel group, B: Blank control group, T: TGF‐β group

**Fig. 2:**
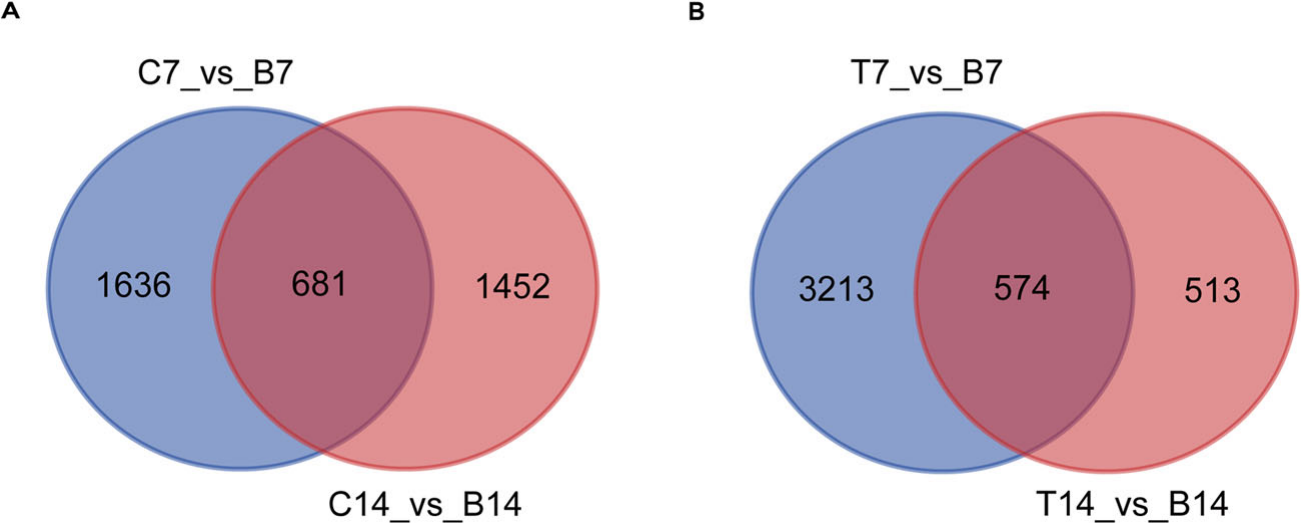
Venn diagram illustrating the differentially expressed genes (DEGs) comparison between 7 days and 14 days. **A** DEGs comparison between C7 vs. B7 and C14 vs. B14 groups using the collagen hydrogel treatment; (**B**) DEGs comparison between T7 vs. B7 and T14 vs. B14 groups using TGF-β treatment. **C** Collagen hydrogel group, B: Blank control group, T: TGF-β group

To gain a global view of the transcriptome profiles during the chondrogenic differentiation of BMSCs mediated by collagen hydrogel, the RNA was isolated from BMSCs cultured in a medium containing either PBS, collagen hydrogel, or TGF‐β for 7 and 14 days and subjected to RNA sequencing. TGF‐β served as a positive control that has been demonstrated to act as an inducer of chondrocyte differentiation. Global gene expression analysis was conducted by comparing transcriptome profiles between the collagen hydrogel group (C) and blank control group (B), as well as between the TGF-β group (T) and blank control group (B). The differentially expressed genes (DEGs) were selected through using fold-change (FC) cutoff criteria of FC > 0.5 or FC < −0.5, in addition to a *p* value < 0.05. Following induction with collagen hydrogel, BMSCs cultured with collagen hydrogel for 7 days (C7) and 14 days (C14) displayed 2317 DEGs compared to those cultured with standard medium. Of these, 1397 genes were upregulated and 920 genes were downregulated in the C7 group. In the C14 group, there were 2133 DEGs consisting of 544 upregulated and 1589 downregulated genes. These findings were illustrated in the volcano plot (Fig. 1A, B). Among these DEGs, a total of 681 unique genes were shared by both time points in the collagen hydrogel treatment groups, compared to the control group, as illustrated in the Venn diagram (Fig. 2A). Similarly, during TGF‐β induction, BMSCs cultured with TGF‐β1 for 7 days (T7) and 14 days (T14) displayed a total of 3787 DEGs compared to those cultured with standard medium. In the T7 group, 2161 genes were upregulated and 1626 genes were downregulated, while in the T14 group, there were 1087 DEGs consisting of 495 upregulated and 592 downregulated genes. These findings are depicted in a volcano plot (Fig. 1C, D). A Venn diagram (Fig. 2B) demonstrated that 574 unique genes were shared by both time points in the TGF‐β1 treatment groups compared to the control group.

### Gene ontology enrichment analysis

**Fig. 3:**
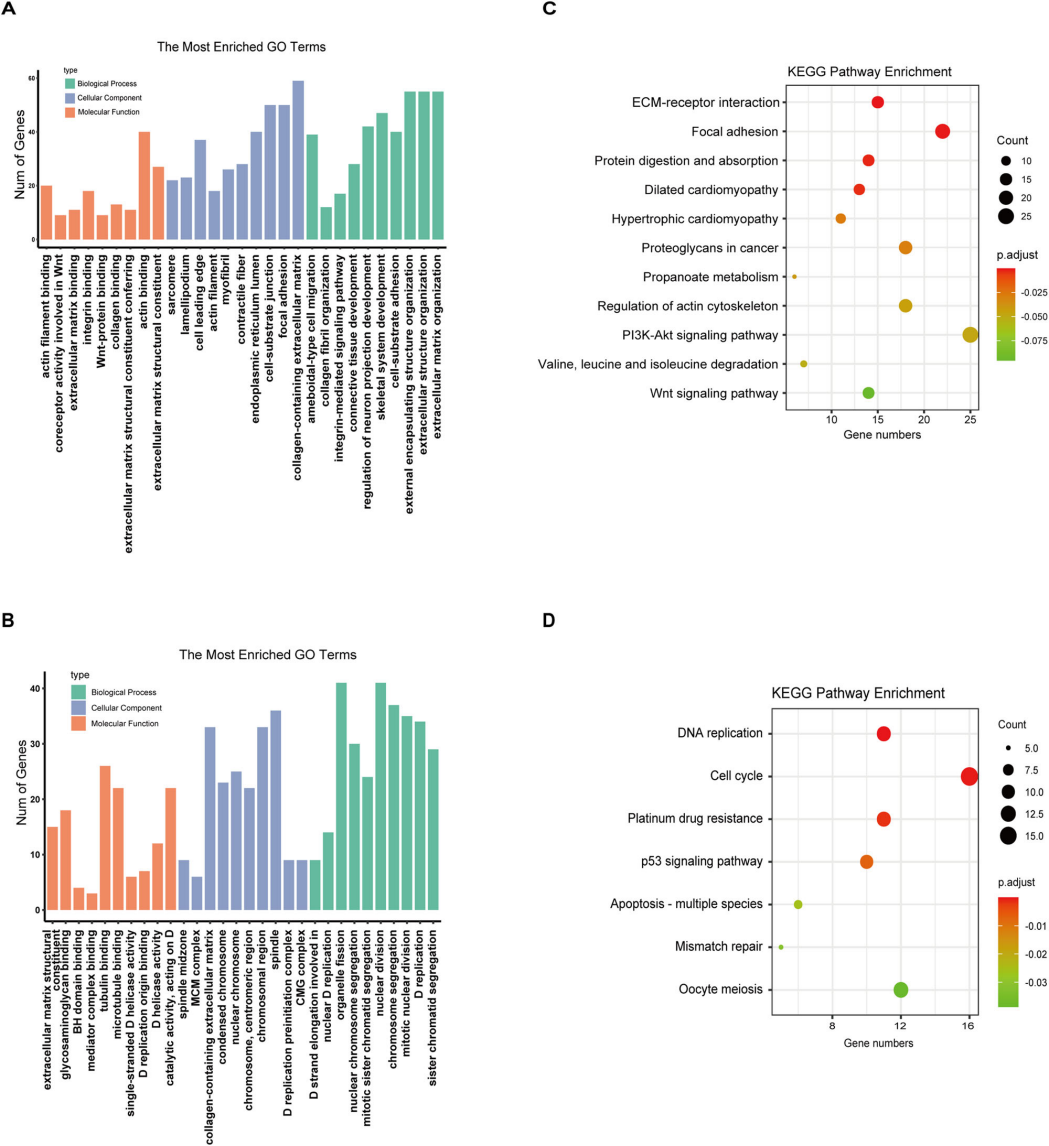
Gene ontology (GO) and KEGG pathway enrichment analysis for differentially regulated genes. (**A**) GO enrichment of DEGs in collagen hydrogel vs. Blank control group; (**B**) GO enrichment of DEGs in TGF‐β vs. Blank control group; (**C**) KEGG pathway enrichment of DEGs in collagen hydrogel vs. Blank control group; (**D**) KEGG pathway enrichment of DEGs in TGF‐β1 vs. Blank control group

We conducted an enrichment analysis of differentially expressed genes during the induction of chondrogenic differentiation in BMSCs using collagen hydrogel, with the Gene Ontology (GO) database. When compared to the control group, GO function enrichment analysis revealed significant enrichment in three categories: molecular function (MF), cellular component (CC), and biological process (BP). Analysis of the biological functions of differently expressed GO categories indicated that DEGs in BP were mainly enriched in extracellular matrix organization, extracellular structure organization, and external encapsulating structure organization, with 55 genes each. Within the CC category, we found that the collagen-containing extracellular matrix was significantly enriched with 59 genes, followed by focal adhesion (50 genes) and cell-substrate junction (50 genes), which were the second and third most highly enriched terms. The top three terms for the most enriched genes in the MF category were actin binding enriched with 40 genes, extracellular matrix structural constituent enriched with 27 genes, and actin filament binding enriched with 20 genes (Fig. 3A). While the DEGs from the TGF‐β1 group vs the blank control group were subjected to GO analysis, and we found that these DEGs were enriched in the organelle fission, chromosome segregation, and nuclear division, which belong to the BP category. And spindle, collagen-containing extracellular matrix, and chromosomal region were terms of the CC category. For the MF category, they were mainly enriched in the tubulin binding, microtubule binding, and catalytic activity, acting on DNA (Fig. 3B). These findings suggest that collagen hydrogel encourages the differentiation of BMSCs into the chondrocytes might by administrating extracellular matrix organization and regulating the interactions between cells and extracellular matrix, which differs from the TGF‐β1induction.

### Kyoto Encyclopedia of Genes and Genomes (KEGG) signaling pathway involved in the chondrogenic differentiation of BMSCs induced by collagen hydrogel

To identify the unique biological significance of DEGs and map them to reference pathways, we conducted pathway enrichment analysis using the Kyoto Encyclopedia of Genes and Genomes (KEGG) database. Figure 3C illustrates 11 enriched pathways involved in the chondrogenic differentiation of BMSCs mediated by collagen hydrogel, including Focal adhesion, Regulation of actin cytoskeleton, Protein digestion and absorption, Dilated cardiomyopathy, ECM-receptor interaction, Hypertrophic cardiomyopathy, Proteoglycans in cancer, Propanoate metabolism, PI3K-Akt signaling pathway, Wnt signaling pathway and Valine, leucine and isoleucine degradation. These KEGG pathways were interrelated rather than independent, with many referring to each other. Among these pathways, the pathway with the most abundant and significant gene enrichment was the focal adhesion signaling pathway, which included 22 genes (Table 2). At the same time, we also analyzed the enrichment pathway of differentially expressed genes between the TGF‐β1 group and the blank control group. Our analysis revealed that these DEGs were predominantly enriched in the Cell cycle, Platinum drug resistance, DNA replication, Apoptosis-multiple species, p53 signaling pathway, Mismatch repair, and Oocyte meiosis (Fig. 3D). Collectively, these findings suggest that collagen hydrogel and TGF-β had different molecular mechanisms in the induction of chondrogenic differentiation. And the focal adhesion signaling pathway may be more specific for the chondrogenic differentiation of BMSCs stimulated by collagen hydrogel than other pathways. Thus, we will focus on the mechanism of collagen hydrogel-induced chondrogenic differentiation of BMSCs for in-depth analysis.

**Table 2:** Genes enriched in focal adhesion signaling pathway

Gene Name	Description	GeneId	C7 vs B7	C14 vs B14
ITGA6	integrin subunit alpha 6	ENSOCUG00000000403	−3.66	−2.89
COL6A2	collagen type VI alpha 2 chain	ENSOCUG00000000409	1.56	−0.74
FLNB	filamin B	ENSOCUG00000000828	−0.86	−2.26
ITGA4	integrin subunit alpha 4	ENSOCUG00000003008	−1.54	−2.54
VCL	vinculin	ENSOCUG00000003163	−0.91	−2.67
CAPN2	calpain-2 catalytic subunit	ENSOCUG00000004612	−0.64	−0.77
PDGFA	platelet derived growth factor subunit A	ENSOCUG00000005660	−1.38	−1.35
TNN	tenascin N	ENSOCUG00000008543	−0.85	−1.85
LAMA3	laminin subunit alpha 3	ENSOCUG00000009869	−1.50	−2.36
FN1	fibronectin 1	ENSOCUG00000010104	−0.75	−2.56
TLN1	talin 1	ENSOCUG00000010203	−1.02	−1.41
ITGA1	integrin subunit alpha 1	ENSOCUG00000011357	−0.96	−1.75
TNC	tenascin C	ENSOCUG00000011500	0.56	−2.61
FLNC	filamin C	ENSOCUG00000011942	−0.65	−1.53
PARVA	parvin alpha	ENSOCUG00000013101	−0.91	−1.62
PDGFD	platelet derived growth factor D	ENSOCUG00000015312	−1.34	−2.93
ACTN1	actinin alpha 1	ENSOCUG00000015449	−1.75	−1.78
ITGAV	integrin subunit alpha V	ENSOCUG00000017626	−0.92	−3.32
ITGA11	integrin subunit alpha 11	ENSOCUG00000017726	−1.22	−3.10
ACTN4	actinin alpha 4	ENSOCUG00000022448	−1.54	−1.97
SPP1	secreted phosphoprotein 1	ENSOCUG00000022536	−1.01	−2.44
COL6A1	collagen type VI alpha 1 chain	ENSOCUG00000025192	1.30	−0.69

### Analysis of genes that are implicated in focal adhesion signaling

**Fig. 4:**
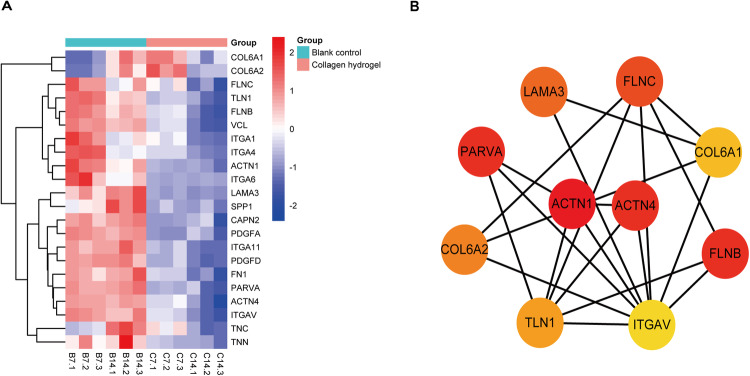
Analysis of key signaling pathway genes. **A** Gene expression levels in the focal adhesion signaling pathway; (**B**) Protein-protein interaction (PPI) network analysis for the genes involved in the focal adhesion signaling pathway

To investigate the expression profile of the focal adhesion signaling pathway components during chondrogenic differentiation induced by collagen hydrogel, the expression levels of genes enriched in the focal adhesion signaling pathway were firstly determined. Their expression pattern was displayed in a heatmap (Fig. 4A). Interestingly, the majority of genes involved in the focal adhesion signaling pathway were notably downregulated during the collagen hydrogel-induced chondrogenic differentiation. Of note, except for three genes, COL6A2, COL6A1, and TNC, all genes related to the focal adhesion pathway showed the same trend at 7 and 14 days after collagen hydrogel induction. Although the expression levels of the three genes (COL6A2, COL6A1, and TNC) increased in 7 days of collagen hydrogel induction compared with the control group, their expressions gradually decreased with the extension of induction time. And their expression was consistently lower than the control group after 14 days of collagen hydrogel induction. To further explore the critical genes that participated in the focal adhesion signaling pathway, we constructed a protein-protein interaction network on these genes enriched in this pathway through the use of the STRING database. As shown in Fig. 4B, we found that the proteins with the most highly connected protein nodes were ACTN1 and ACTN4, interacting with more partners than the average. Therefore, ACTN1 and ACTN4 were identified as critical genes within the whole network. Of note, ACTN1 and ACTN4 were also crucial factors regulating the actin cytoskeleton signaling pathway. Besides, combining gene expression profiles, expression of ACTN1 and ACTN4 was dramatically decreased at 7 and 14 days after collagen hydrogel induction. Interestingly, ACTN1 and ACTN4 belong to isoforms of α-actinin. Hence, these results indicated that the two α-actinin isoforms, ACTN1 and ACTN4, may have a crucial function in collagen hydrogel-induced chondrogenic differentiation.

### The correlation between N6-methyladenosine RNA methylation regulation factors

**Fig. 5:**
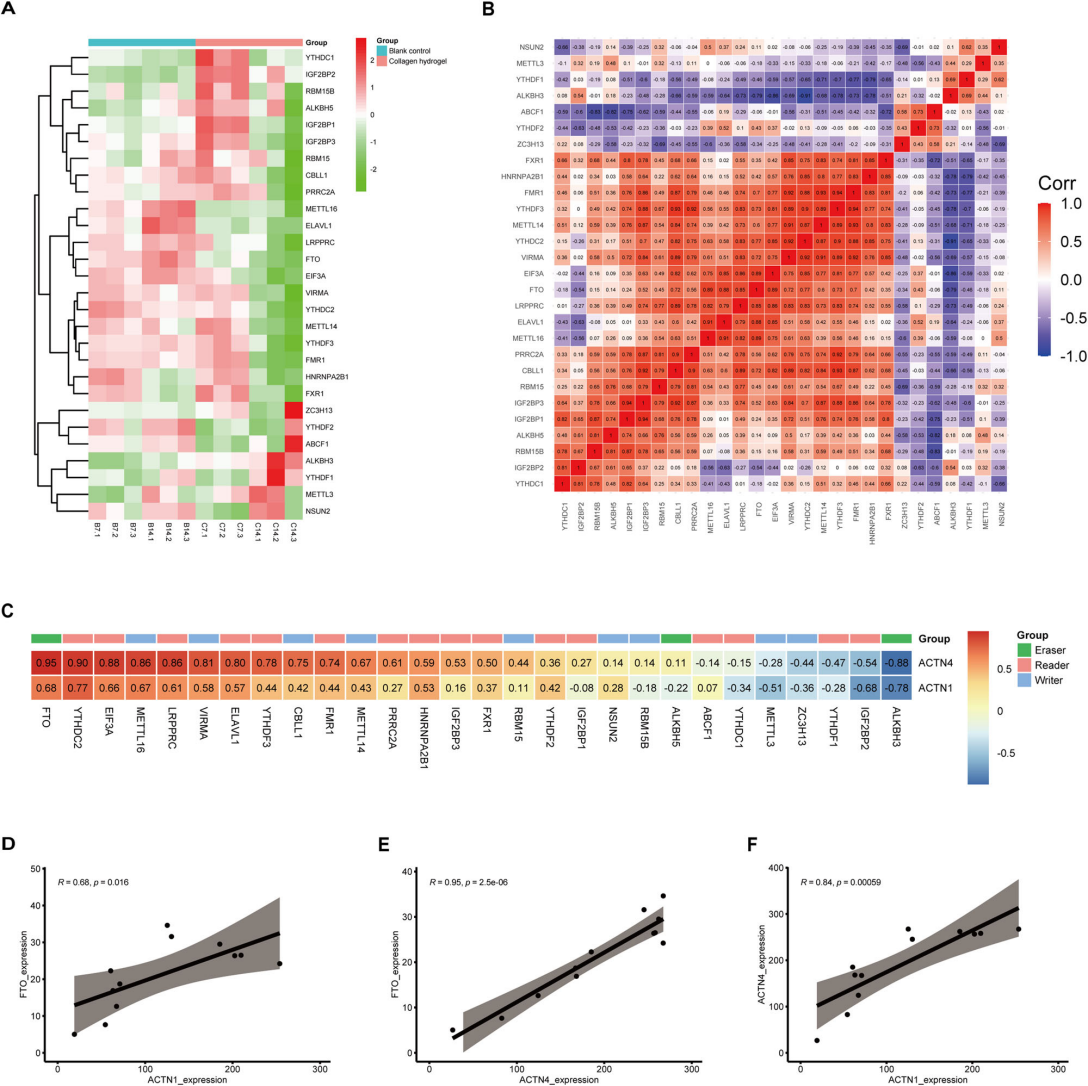
Correlation of 28 N6-Methyladenosine modifiers. **A** Gene expression levels of 28 modulators involved in N6-Methyladenosine RNA methylation; (**B**) The correlation among 28 m6A modification regulators; (**C**) The correlation of two essential genes and 28 m6A modification regulators; (**D**) The correlation analysis between FTO and ACTN1; (**E**) The correlation analysis between FTO and ACTN4; (**F**) The correlation analysis between ACTN1 and ACTN4

Given the importance of m6A regulation in the differentiation of stem cells [14–16], we hypothesized that such modifications could have a critical function in the chondrogenic differentiation process of BMSCs. Hence, we investigated whether m6A regulation factors were associated with the chondrogenic differentiation of BMSCs mediated by collagen hydrogel. Firstly, the expression levels of 28 factors involved in the regulation of N6-methyladenosine RNA methylation, including nine writers (METTL3, METTL14, METTL16, CBLL1, NSUN2, VIRMA, RBM15, ZC3H13, and RBM15B), three erasers (FTO, ALKBH5, and ALKBH3), and 16 readers (YTHDC2, YTHDF1, YTHDF2, YTHDF3, YTHDC1, IGF2BP1, IGF2BP2, HNRNPA2B1, IGF2BP3, PRRC2A, FMR1, LRPPRC, FXR1, EIF3A, ABCF1, and ELAVL1), were analyzed during the chondrogenic differentiation of BMSCs mediated by collagen hydrogel. As shown in Fig. 5A, the expression of METTL16, ELAVL, LRPPRC, and FTO was consistently decreased in BMSCs after 7 and 14 days of collagen hydrogel induction, while YTHDF2, ABCF1, ALKBH3, and YTHDF1 expressions were increased after collagen hydrogel induction, the expression of the remaining m6A regulators was elevated during early chondrogenic differentiation and decreased in the later period of chondrogenic differentiation. Furthermore, we conducted a Pearson correlation test to examine the correlation between 28 m6A regulatory factors and observed that seven regulators, including NSUN2, METTL3, YTHDF1, ALKBH3, ABCF1, YTHDF2, and ZC3H13, were negatively related with most of the other regulators. Of note, “eraser” FTO exhibited a positive correlation with EIF3A (with a correlation coefficient of 0.89) and ELAVL1 (with a correlation coefficient of 0.88) but a negative correlation with ALKBH3 (with a correlation coefficient of −0.79), IGF2BP2 (with a correlation coefficient of −0.54), and YTHDF1(with a correlation coefficient of −0.46). Meanwhile, the “writer” METTL14 was positively correlated with FMR1 (with a correlation coefficient of 0.93) and VIRMA (with a correlation coefficient of 0.91). Interestingly, we also noted that “reader” YTHDF3 displayed the highest correlation with FMR1 (its correlation coefficient up to 0.94) among all m6A RNA methylation regulation factors (Fig. 5B). Consequently, a significant number of regulators responsible for the regulation of m6A RNA methylation exhibited robust correlations with each other.

**Fig. 6:**
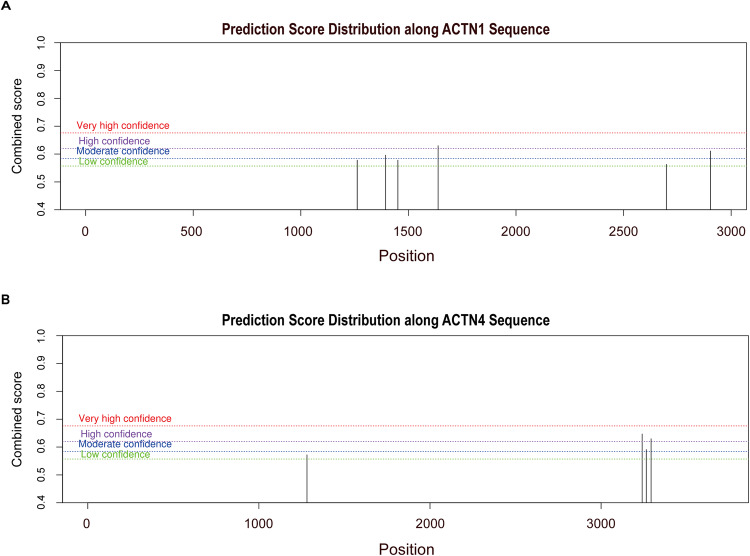
Prediction of m6A site(s) for key genes. (**A**) Prediction of m6A sites in the ACTN1 gene using SRAMP. (**B**) Prediction of m6A sites in the ACTN1 gene using SRAMP

Given that m6A is the main internal modification of mRNA fate regulation, we further studied the relationship between m6A regulatory factors and two key genes (ACTN1 and ACTN4) involved in chondrogenic differentiation. As shown in Fig. 5C, among all the m6A regulatory factors, m6A demethylase FTO displayed the strongest positive correlation with ACTN1 and ACTN4. Thus, we further exhibited the relationship between two key genes (ACTN1 and ACTN4) and the m6A demethylases FTO through the level of gene expression (Fig. 5D–F). In addition, we also predicted the m6A sites of ACTN1 and ACTN4 sequences through the SRAMP database (http://www.cuilab.cn/sramp). The results showed that ACTN1 contained 6 m6A binding sites (Fig. 6A), including one highly confident m6A site (Table 3), while ACTN4 contained 4 m6A binding sites (Fig. 6B), including 2 highly confident m6A sites (Table 4), indicating that ACTN1 and ACTN4 may undergo m6A modification. Altogether, we concluded that m6A regulatory factor FTO might participate in the regulation of ACTN1 and ACTN4 mRNA stability or transcription, which have significant functions in the progression of collagen hydrogel-induced chondrogenic differentiation of BMSCs.

**Table 3:** Prediction score distribution along ACTN1 sequence

Position	Sequence context	Structural context	Score (binary)	Score (knn)	Score (spectrum)	Score (combined)	Decision
1262	GAGGCCAUGCUGCGGCAGAAGGACUACGAGACGGCCACGCUGUCG	N/A	0.655	0.644	0.464	0.578	m6A site (Low confidence)
1394	GCGCAGGAGCUCAAUGAGCUGGACUAUUACGACUCACCCAGUGUC	N/A	0.722	0.707	0.408	0.596	m6A site (Moderate confidence)
1451	CAAAAGAUCUGUGACCAGUGGGACAAUCUGGGGGCCCUAACCCAG	N/A	0.605	0.731	0.521	0.578	m6A site (Low confidence)
1638	ACACCAUCGAGGAGAUCCAGGGACUGACCACAGCCCACGAGCAGU	N/A	0.776	0.733	0.417	0.63	m6A site (High confidence)
2699	GAUGCCGUGCCCGGUGCCCUGGACUACAUGUCCUUCUCCACGGCG	N/A	0.591	0.718	0.506	0.563	m6A site (Low confidence)
2904	AAGCCUCCCAGCCCACAAAGUGACAGUUUACAAAAUUAUUUUCUG	N/A	0.674	0.787	0.502	0.611	m6A site (Moderate confidence)

**Table 4:** Prediction score distribution along ACTN4 sequence

Position	Sequence context	Structural context	Score (binary)	Score (knn)	Score (spectrum)	Score (combined)	Decision
1281	GAGGCCAUGCUGAGGCAGCGGGACUACGAGACGGCCACCCUGGCG	N/A	0.618	0.665	0.499	0.572	m6A site (Low confidence)
3240	ACCCUGGCCUUGCUUUGUCUGGACUCACGGCUCAGAUUUUCUAAG	N/A	0.708	0.846	0.539	0.647	m6A site (High confidence)
3264	UCACGGCUCAGAUUUUCUAAGGACCAAAAAACACAACAAAAACCC	N/A	0.632	0.645	0.529	0.591	m6A site (Moderate confidence)
3292	AAACACAACAAAAACCCAACAGACUUACAAAAUCUAAAAAUUUCC	N/A	0.74	0.744	0.464	0.63	m6A site (High confidence)

### Verification of expression of critical m6A regulatory factor and essential genes involved in focal adhesion signaling

**Fig. 7:**
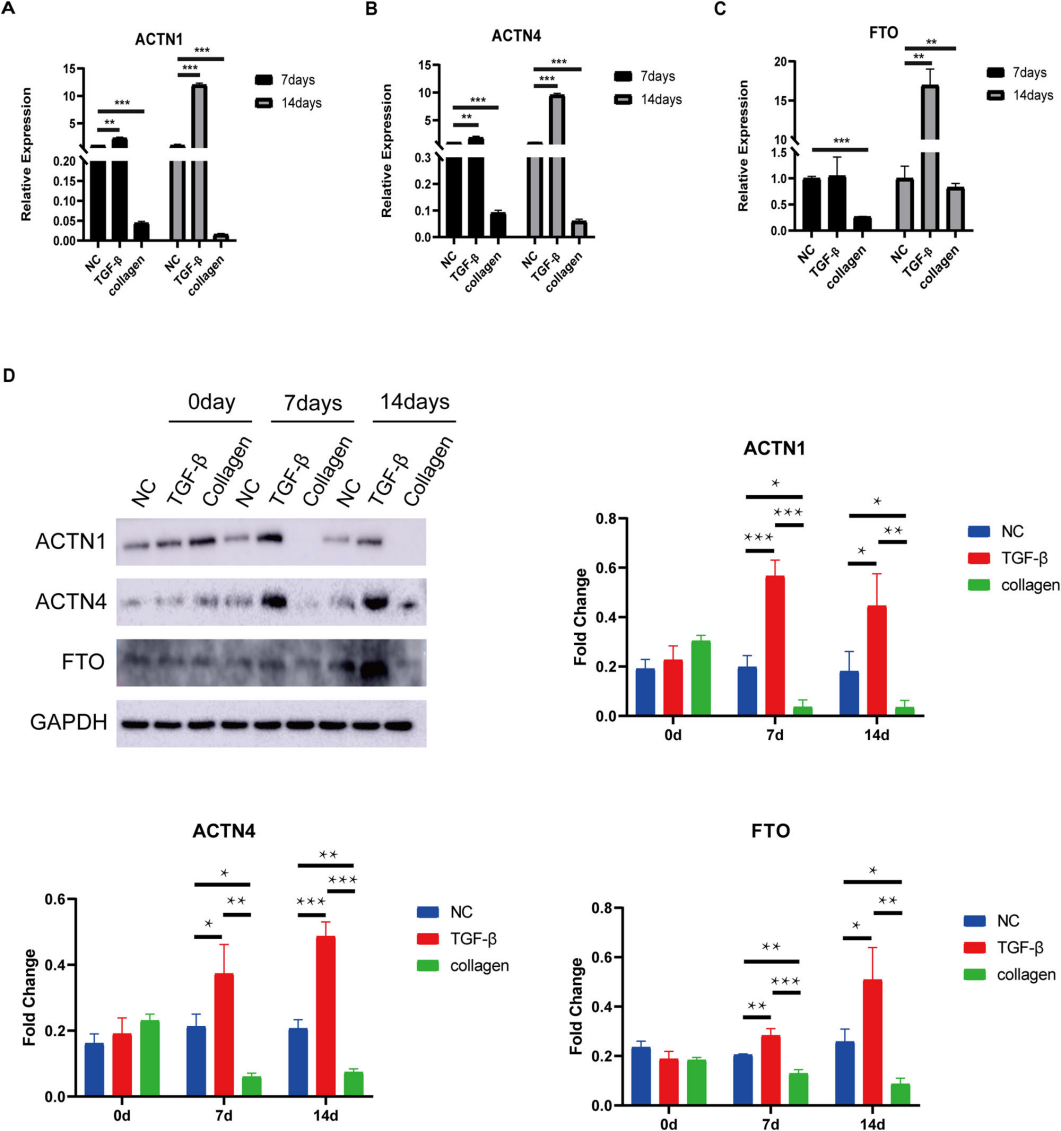
Verification of expression levels of key genes and key m6A modification regulator. **A** RT-qPCR analysis confirmed the expression levels of ACTN1. **B** RT-qPCR analysis confirmed the expression levels of ACTN4. **C** RT-qPCR analysis confirmed the expression levels of FTO. These data represent the means ± SD of three independent experiments; *p < 0.05, **p < 0.01, and ***p < 0.001. **D** Western blot analysis was used to measure the protein levels of ACTN1, ACTN4, and FTO. Representative images from one of three independent experiments are shown, and the quantitative data are presented as the means ± SD; *p < 0.05, **p < 0.01, and ***p < 0.001

To verify the relative abundance of the m6A regulatory factor FTO and two key genes involved in focal adhesion signaling, ACTN1 and ACTN4, in the BMSCs after 7 and 14 days of collagen hydrogel induction, we implemented an RT-qPCR assay. The RT-qPCR results illustrated that the relative expression levels of ACTN1, ACTN4 and FTO were considerably decreased in the collagen hydrogel induction group after 7 and 14 days when compared to those observed in the blank control group, while their expression had a weak upregulation in the TGF-β group (Fig. 7A–C). The western blot analysis revealed that the protein levels of ACTN1, ACTN4, and FTO were reduced in the collagen hydrogel induction group 7 and 14 days after treatment when compared with those in the blank control group. While their expression displayed meaningful upregulation in the TGF-β group compared to that in the blank control group (Fig. 7D). These findings provide evidence supporting the existence of positive correlations between FTO and ACTN1 as well as ACTN4, respectively. Overall, these data indicated that m6A regulatory factors, FTO, might regulate ACTN1 and ACTN4 levels, thereby accelerating collagen hydrogel-induced chondrogenic differentiation of BMSCs.

## Discussion

Articular cartilage defects are a common medical and social problem. BMSCs have been considered a feasible and effective treatment for cartilage damage because of their potential of differentiation into chondrogenic lineage under the action of a suitable inducer and easy harvesting from various kinds of tissues. Our previous investigations have found that collagen hydrogel can effectively facilitate the differentiation of BMSCs into chondrocytes without any other inducers and is promising for treating cartilage defects. However, the molecular mechanism of its induction is not clear. Thus, identification and analysis of genes with differential expression during chondrogenic differentiation of BMSCs induced by collagen hydrogel could provide a thorough comprehension of the intricate details of chondrogenic commitment, as well as their potential therapeutic efficacy in treating articular cartilage defects.

In this investigation, we demonstrated that during collagen hydrogel-induced chondrogenic differentiation of BMSCs, a substantial number of genes showed significant upregulation or downregulation. Specifically, after 7 days of differentiation, 1397 genes were found to be upregulated while 920 genes were downregulated. Meanwhile, at 14 days of differentiation, the number of upregulated genes decreased to 544 while the number of downregulated genes increased to 1589. These findings suggest that the dynamic changes in gene expression patterns over time during this process and could potentially have a crucial role in the chondrogenic differentiation of BMSCs induced by collagen hydrogel. Furthermore, these genes with significant alterations in expression may be closely involved in the process of chondrogenesis.

Bioinformatics analysis revealed that differential genes between the collagen hydrogel group and control group were substantially enriched in various signaling pathways, such as Focal adhesion signaling, Regulation of actin cytoskeleton, ECM-receptor interaction, and PI3K-Akt signaling pathway. These pathways crosstalk with each other and have been recognized as the critical pathways that are potentially involved in the chondrocyte differentiation of BMSCs [17], with the focal adhesion signaling pathway being particularly noteworthy. This pathway serves as a biochemical signaling hub that enables the binding between the cytoskeleton and mediates the connection of the actin cytoskeleton of chondrocytes to the extracellular matrix, thereby triggering intracellular signaling cascades that are ultimately transmitted to the transcriptional machinery within the nucleus of the cell [18, 19]. Our current study revealed that a significant proportion of the genes with differential expression were enriched in the focal adhesion signaling pathway, as determined by their *p* values and the number of genes associated with the enrichment, during the process of collagen hydrogel-induced chondrogenic differentiation of BMSCs. Increasing evidence uncovered that the focal adhesion signaling pathway has an essential role in stem cell differentiation [20]. The dynamic changes in focal adhesions could lead to different lineage determinations of stem cells. For example, the more significant numbers of focal adhesions were favored for osteogenic differentiation of BMSCs, whereas fewer focal adhesions could encourage stem cells to differentiate into chondrocytes [21]. Shin et al. [22] found that inhibiting the activity of focal adhesion complexes can prevent the loss of chondrogenic phenotypes and the dedifferentiation of chondrocytes. Thus, control of genes associated with focal adhesions has a profound impact on controlling the chondrogenic differentiation of BMSCs. Interestingly, our findings revealed that the majority of genes participating in the focal adhesion signaling pathway were notably downregulated during the collagen hydrogel-induced chondrogenic differentiation, implying collagen hydrogel stimulation resulted in fewer focal adhesions, thereby promoting the chondrogenic potential of BMSCs, which is consistent with the notion that fewer focal adhesion attachments were beneficial to chondrogenesis [21, 23]. Except for focal adhesion signaling, regulation of actin cytoskeleton also has a fundamental role in chondrogenesis regulation. Lim et al. [24] revealed that perturbing the actin cytoskeleton using cytochalasin-D can enhance chondrogenesis in mesenchymal stem cells obtained from chicken wing buds. Our data revealed that many DEGs were also enriched in the regulation of actin cytoskeleton signaling pathway. Notably, the expression levels of these enriched genes were significantly declined in the collagen hydrogel-induced group, indicating that the treatment of collagen hydrogel gave rise to the alteration in cytoskeletal configuration, thereby promoting chondrocyte differentiation of BMSCs. Thus, in conjunction with our findings, these data offer additional evidence for the critical functions of the focal adhesion and actin cytoskeleton signaling pathways in collagen hydrogel-induced chondrogenic differentiation of BMSCs. While our study primarily focused on the focal adhesion and actin cytoskeletal signaling pathways, it is essential to acknowledge that other potential mechanisms may also contribute to collagen hydrogel-induced chondrogenesis, such as ECM-receptor interaction and PI3K-Akt signaling pathway. Exploring these alternative pathways and their interplay with the focal adhesion and actin cytoskeletal signaling pathways could provide valuable insights into the complex regulatory network underlying chondrogenesis induced by collagen hydrogel and potentially contribute to developing more effective strategies for enhancing chondrogenesis in tissue engineering applications. However, further studies are needed to elucidate the specific roles and contributions of these alternative pathways, as well as their crosstalk with the focal adhesion and actin cytoskeletal signaling pathways.

In the present research, we found that ACTN1 and ACTN4 were hub genes of focal adhesion signaling and exhibited significant downregulation during chondrocyte differentiation of BMSCs induced by collagen hydrogel. ACTN1 and ACTN4 belong to non-muscle isoforms of the family of α-actinin. Both of these isoforms are critically involved in preserving the structural integrity and stability of the cytoskeleton. Any alterations in their expression levels may result in physiological and pathological changes in humans [25–27]. ACTN1 and ACTN4 commonly form a heterodimer in various cell types, and the dimer interacts with common or distinct ligands [27, 28], indicating particular biological roles. The research has demonstrated the significant involvement of both ACTN1 and ACTN4 in the regulation of cytoskeleton organization, cell adhesion, and cell motility [29, 30]. Interestingly, we also observed that ACTN1 and ACTN4 were crucial genes regulating the actin cytoskeleton signaling pathway during chondrogenic differentiation of BMSCs induced by collagen hydrogel. Indeed, ACTN1 and ACTN4 have been demonstrated to effectively crosslink actin filaments in the cytoskeleton of non-muscle cells, which plays a pivotal role in sustaining the stability of structure and strength of the cytoskeleton by connecting actin filaments in a parallel orientation [31]. Thus, we speculated that ACTN1 and ACTN4 were likely to participate in and simultaneously regulate focal adhesion and actin cytoskeleton signaling pathways, which play a pivotal role in regulating chondrogenesis. According to recent studies, modifications in focal adhesion and reorganization of the cytoskeleton can significantly influence cell morphology. The changes in cellular shape and cytoskeletal structure, which are closely associated with cell morphology, also have critical effects on MSCs differentiation. It has been reported that chondrogenesis is highly dependent on the cellular shape and is promoted by having a rounded cell morphology [21]. Previous studies have demonstrated that MSCs, when plated on RGD (Arginine-Glycine-Aspartic Acid) polypeptide-modified surfaces, undergo spreading. In contrast, when these cells were seeded on the surfaces coated with RGE (Arginine-Glycine-Glutamic Acid) polypeptides modifications that inhibit focal adhesion attachment, they adopted a rounded shape. It was also observed that MSCs plated on RGD surfaces had lower mRNA levels for collagen II and aggrecan, indicating reduced chondrogenesis [23, 32]. It exemplifies that ACTN1 and ACTN4 may influence the shape of BMSCs by regulating the focal adhesion and actin cytoskeleton signaling pathways, thereby accelerating their chondrogenic potential under the stimulation of collagen hydrogel.

M6A methylation is a critical post-transcriptional modification that plays a significant role in regulating numerous RNA metabolism functions, such as pre-mRNA splicing, mRNA stability, translation, and transport. These processes are implicated in regulating several key cellular activities like cell proliferation, cell cycle regulation, cell fate decision, and differentiation [33, 34]. Growing evidence suggests that m6A methylation can efficiently control the proliferation and differentiation of stem cells via regulating the expression of genes linked to pluripotency, expansion, or lineage-specific genes, implying its pivotal role in facilitating the differentiation of stem cells [35]. In our study, we found that FTO displayed the strongest positive correlation with ACTN1 and ACTN4. Being an m6A eraser, FTO is known to possess demethylase activity for m6A [36]. Existing evidence suggests that FTO has a significant impact on the regulation of RNA stability, splicing, export, and metabolism by modulating RNA m6A levels in eukaryotes [37]. Thus, we speculated that FTO positively regulated the expression of ACTN1 and ACTN4 might by modulating the m6A level of ACTN1 and ACTN4 mRNA, thereby enhancing the chondrogenic differentiation of BMSCs. A growing body of evidence has demonstrated the critical functions of FTO in the differentiation process of BMSCs [38]. Yuhang Cao et al. [39] revealed that the knockout of FTO in adult neural stem cells facilitates the differentiation of neurons and increases the proliferation of adult neural stem cells. Our findings are consistent with these observations, showing that expression of FTO was significantly decreased during the chondrocyte differentiation of BMSCs induced by collagen hydrogel. Combined with previous research findings, our results indicated that collagen hydrogel might regulate focal adhesion and actin cytoskeletal signaling pathways through down-regulation of ACTN1 and ACTN4 mRNA mediated by m6A demethylase FTO, ultimately driving chondrogenic differentiation of BMSCs. However, further investigations are required to validate our hypothesis and elucidate the precise connection between FTO and collagen hydrogel-stimulated chondrocyte differentiation, as well as the molecular mechanisms that underlie the regulation of ACTN1 and ACTN4 by FTO.

In conclusion, our findings suggest that collagen hydrogel possesses the properties of cartilage induction and could foster the chondrocyte differentiation of BMSCs. The genomics and bioinformatics analyses demonstrated that focal adhesion and actin cytoskeletal signaling pathways are instrumental in mediating the mechanical induction of chondrogenic differentiation by collagen hydrogel. ACTN1 and ACTN4, two isoforms of α-actinin, may play essential roles in regulating the chondrogenic differentiation of BMSCs. Furthermore, our research findings indicate a strong relationship between the expression levels of two isoforms and the m6A demethylase FTO. These results offer novel insights into the underlying molecular mechanisms implicated in the collagen hydrogel-induced chondrogenic differentiation of BMSCs, which could serve as a promising therapeutic strategy for treating articular cartilage defects.

## Data Availability

The data used to support the findings of this study are available from the corresponding author upon request.
